# Extreme bone lengthening by bone transport with a unifocal tibial corticotomy: a case report

**DOI:** 10.1186/s12891-019-2927-z

**Published:** 2019-11-20

**Authors:** Hongjie Wen, Huagang Yang, Yongqing Xu

**Affiliations:** 1 Department of Orthopedic Surgery, The Fourth Affiliated Hospital of Kunming Medical University, 176 Qinnian Road, Wuhua District, Kunming, Yunnan 650021 People’s Republic of China; 2 Department of Orthopedic Surgery, 920th Hospital of Joint Logistics Support Force, 212 Daguan Road, Xi Shan District, Kunming, Yunnan 650031 People’s Republic of China

**Keywords:** Ilizarov technique, Tibial nonunion, Bone defects, Bone transport

## Abstract

**Background:**

Bone transport is used for the treatment of extensive limb bone defects. The application of ring or unilateral external fixators combined with single or double corticotomy are well documented; however, there are few cases adopting a single corticotomy to repair bone defects > 24 cm.

**Case presentation:**

The present case study describes an 18-year-old male, who was involved in a traffic accident and was diagnosed with open fracture of the right tibia. The patient received emergency surgery in a local hospital and was transferred to The Second People’s Hospital of Yunnan for further treatment 3 months later. The patient was diagnosed with fracture nonunion and infection following admission. Complete debridement was performed three times to control the infection. The infection was resolved after 26 days and the 24.5 cm massive tibia defect remained the biggest challenge. The bone transport technique involving a unilateral external fixator and single corticotomy was employed to treat the bone defect. Docking site union was achieved and bone consolidation was complete 40 months after corticotomy. The external fixator was subsequently removed. The bone healing index was 1.6 months/cm. The Association for the Study and Application of the Method of Ilizarov criteria (ASAMI) revealed a good functional and bone repair result. Similarly, Knee Society Score (KSS) yielded good result and the The Lower Extremity Functional Scale (LEFS) was 65. A total of 45 months after injury, the patient was able to walk painlessly without ambulatory assistive devices and resumed daily activities successfully. Eighteen months after the bone and soft tissue wound have healed, the SF-36 score was 86, and the LEFS was 70.

**Conclusion:**

To the best of the authors’ knowledge, the present study described the longest bone defect repair performed using bone transport with single level corticotomy.

## Background

Tibial defects > 5 cm secondary to high-energy trauma or debridement following nonunion pose a significant challenge in orthopedic surgery [1]. Methods for treating massive bone defects include bone transport, Masquelet technique and bone grafts with or without vascular pedicle. Each method has its advantages and disadvantages [2–6]. The Ilizarov technique is currently the gold standard treatment for large segmental defects as it does not require large amounts of soft tissue and autologous bone grafts. Furthermore, infections are adequately controlled and bone and soft tissue defects may be repaired at the same time [7]. According to current literature, single and double osteotomies have been performed for bone transfers up to 22 and 14.5 cm in length, respectively [8–10].

The present case study demonstrated the application of unilateral external fixator and single osteotomy for the treatment of infected tibial bone defects caused by severe trauma.

## Case presentation

**Fig. 1:**
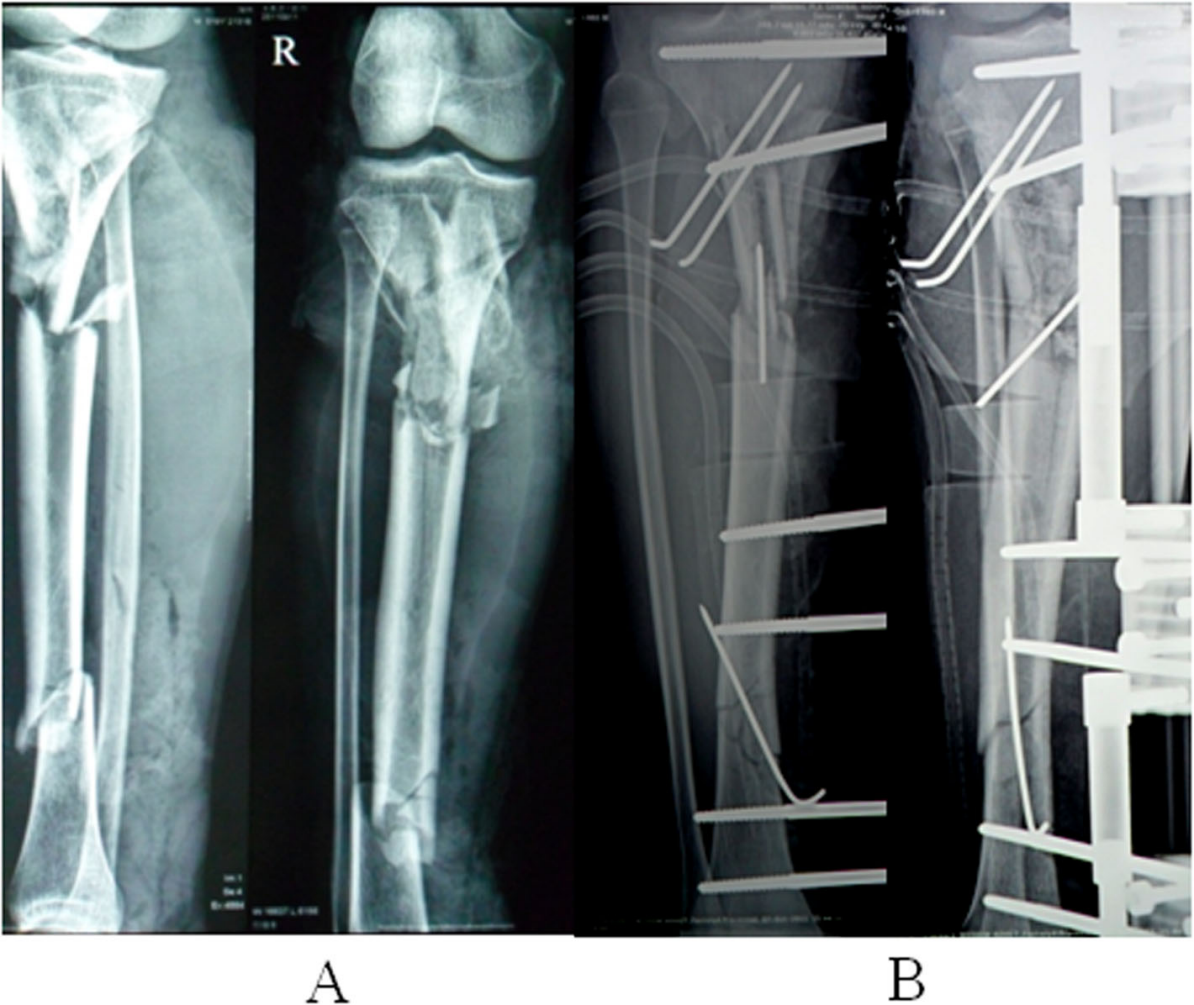
X-ray (**a**) at the time of injury and (**b**) following emergency debridement in the local hospital

In September 2014, an 18-year-old, non-smoking male was diagnosed with an open fracture of the right tibia, Gustilo’s classification grade IIIB [11] following a traffic accident. The patient weighed 65 kg. Emergency debridement was performed at the local hospital following injury (Fig. 1a and b). The fracture and wound had not healed 3 months after injury. Copious pus was drained and the patient was transferred The Second People’s Hospital of Yunnan for further treatment.

**Fig. 2:**
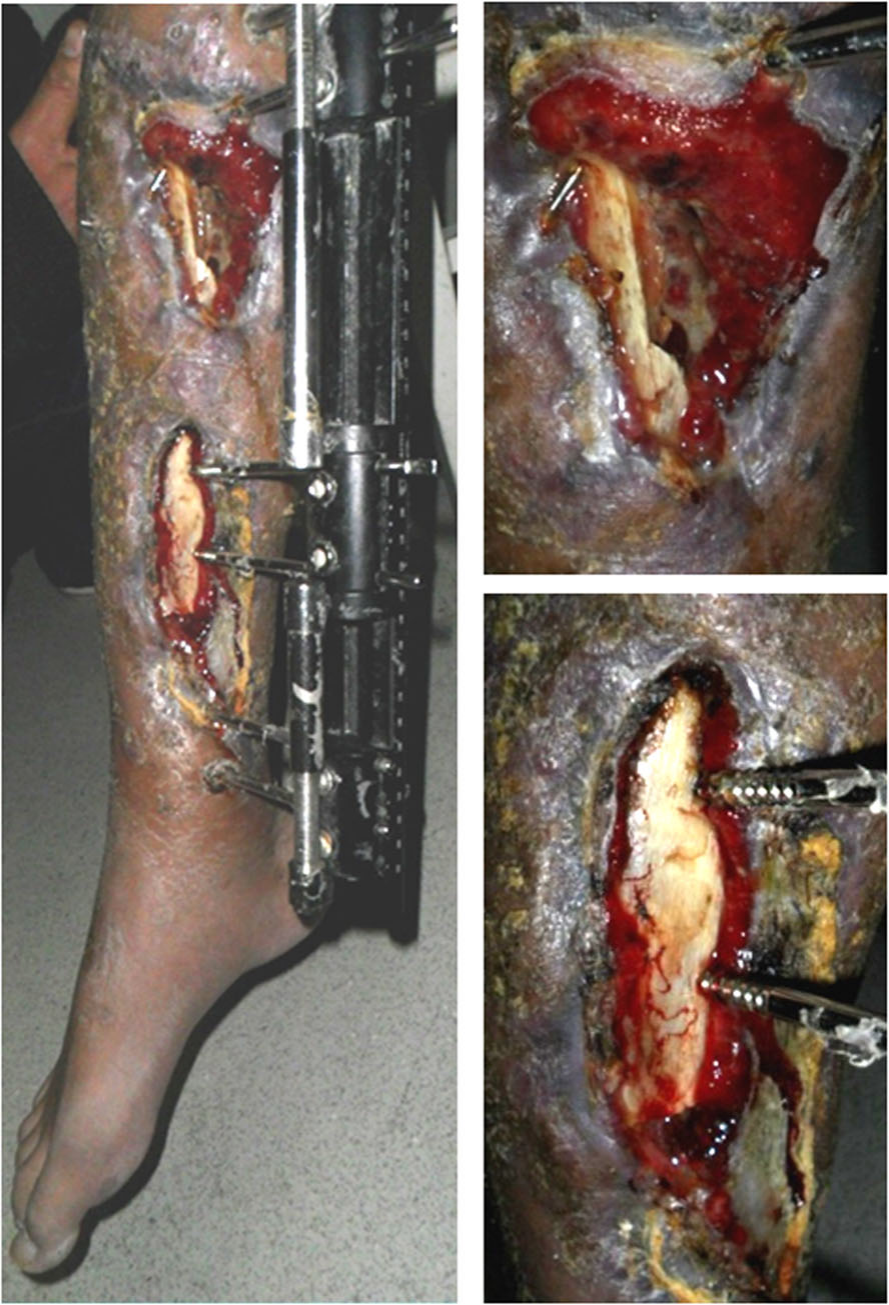
A 4x10cm incision was observed on the anterior medial side of the upper calf and a 3x12cm wound was seen on the anterior medial side of the lower leg. Significant foot drop was observed

The patient was admitted to The Second People’s Hospital of Yunnan 97 days after the injury. A physical examination of the patient’s right calf revealed a soft tissue defect and exposed tibia. A 4 × 10 cm incision was observed on the anterior medial side of the upper calf and a 3 × 12 cm-sized wound was seen on the anterior medial side of the lower leg. Significant foot drop was observed. The patient experienced hypoesthesia on the lateral side of right calf and the dorsal surface of the foot; however, normal sensation was felt on the plantar surface of the foot and the dorsalis pedis artery appeared normal. The range of motion of the knee was 0–80°, and ankle extension and flexion were 10 and 20°, respectively. Level III myodynamia was observed in the pretibial muscle and the triceps surae (Fig. 2).

**Fig. 3:**
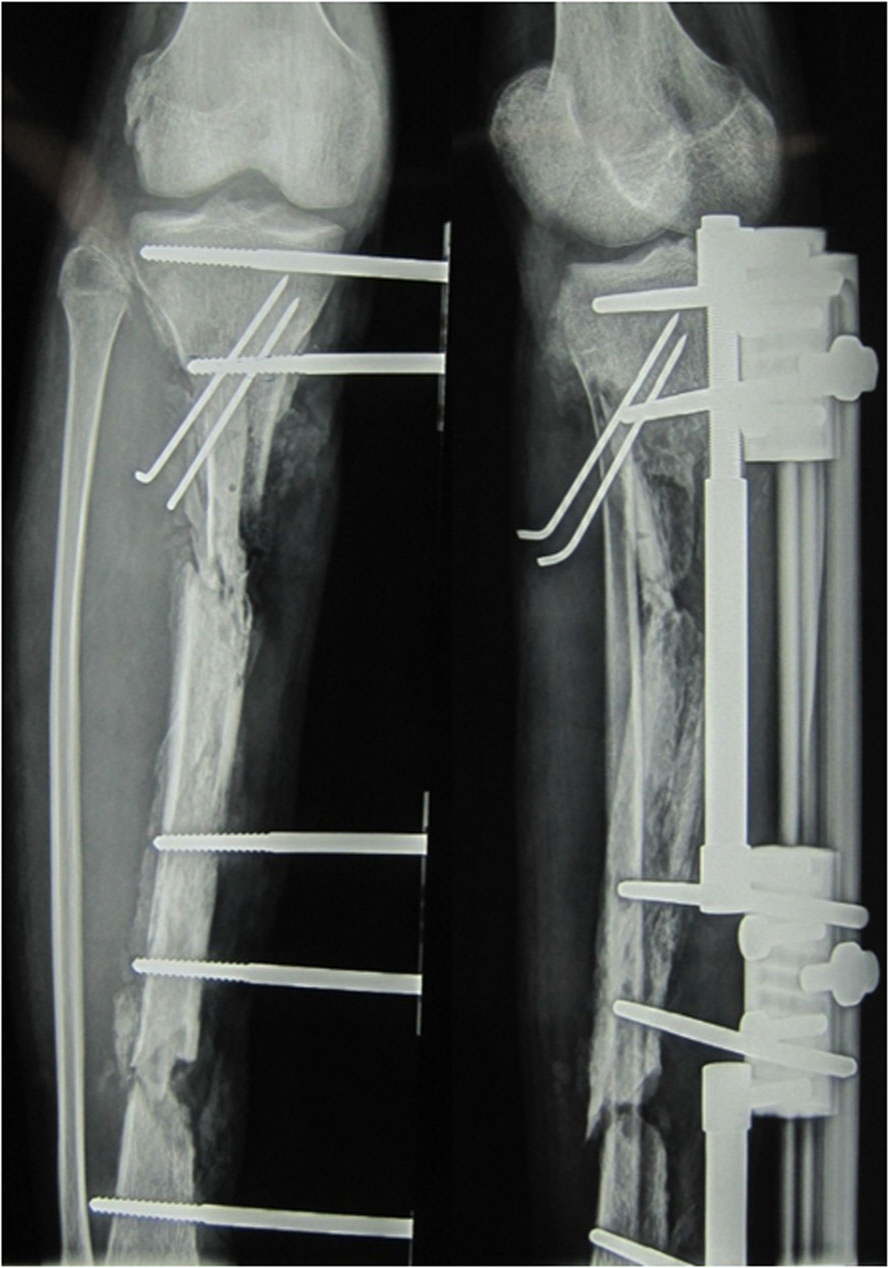
Preoperative X-ray on the day of admission at The Second People’s Hospital of Yunnan demonstrating the necrotic bone

Following discussion with the patient, a limb salvage protocol was adopted in favor of amputation. The treatment plan consisted of two stages. The first step was to treat the infection by performing repeated debridement, replacing the external fixation, vacuum sealing drainage (VSD) and local and systemic application of antibiotics. In the second stage, the bone transport technique was used to repair the bone defect, and rehabilitation exercise was performed to restore the function of the affected limb as much as possible. The patient was informed about the treatment plan and the associated risks. A series of examinations were performed to assess the tibial infection. An X-ray revealed necrosis and hyperplasia in the middle of tibia (Fig. 3). Single-photon emission computed tomography showed radionucleotide accumulation in the midtibial region and computed tomography angiography confirmed that the anterior and posterior tibial arteries were normal. Laboratory investigations were as follows: i) C-reactive protein, 21.6 mg/l: ii) erythrocyte sedimentation rate, 35 mm/h; iii) white blood cell count, 16.47 × 10^9^/l; and iv) Neutrophil percentage, 84.51[50–70]. The final diagnosis was an infected right tibial nonunion with a soft tissue defect.

**Fig. 4:**
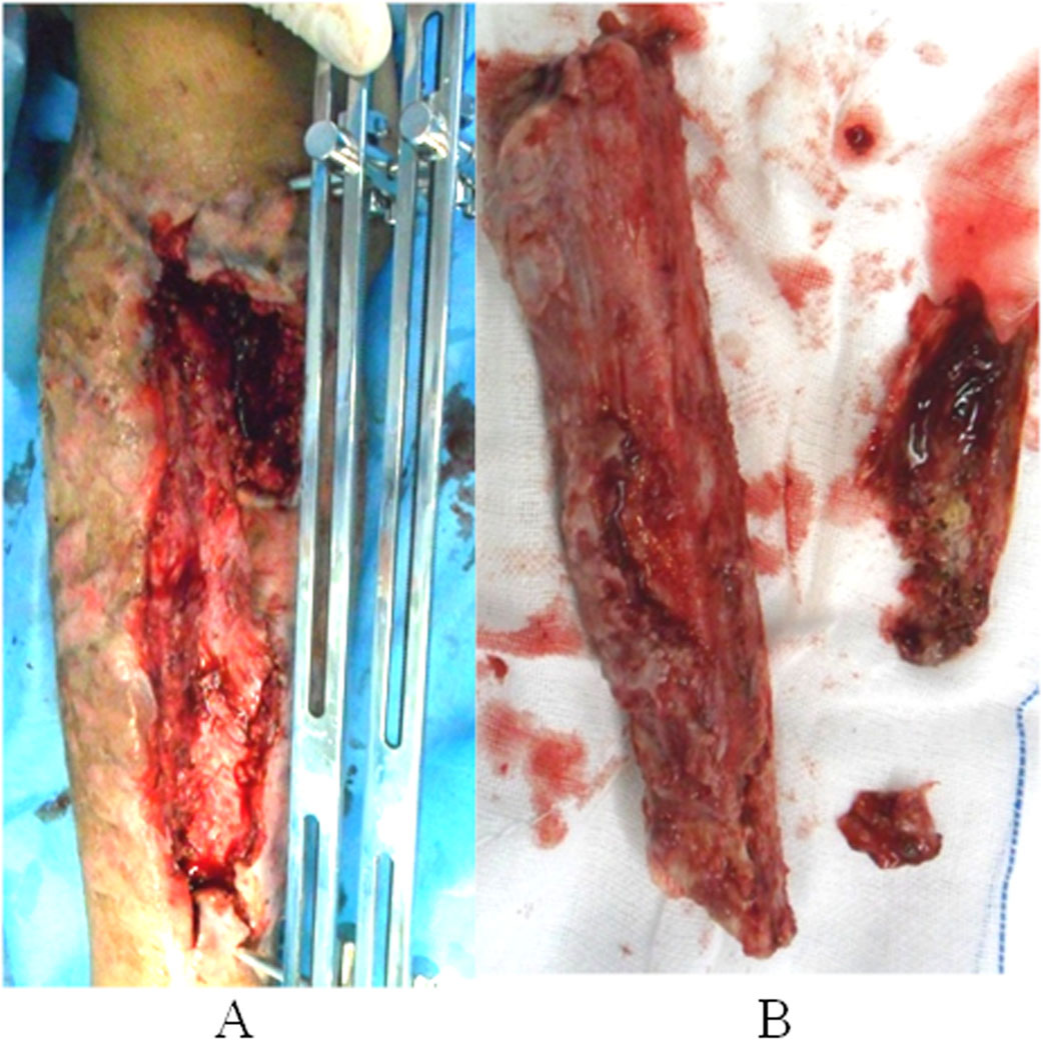
The first debridement procedure. **a** Significant purulent necrotic tissue was attached to the necrotic bone. **b** No bleeding was observed in the bone cortex

**Fig. 5:**
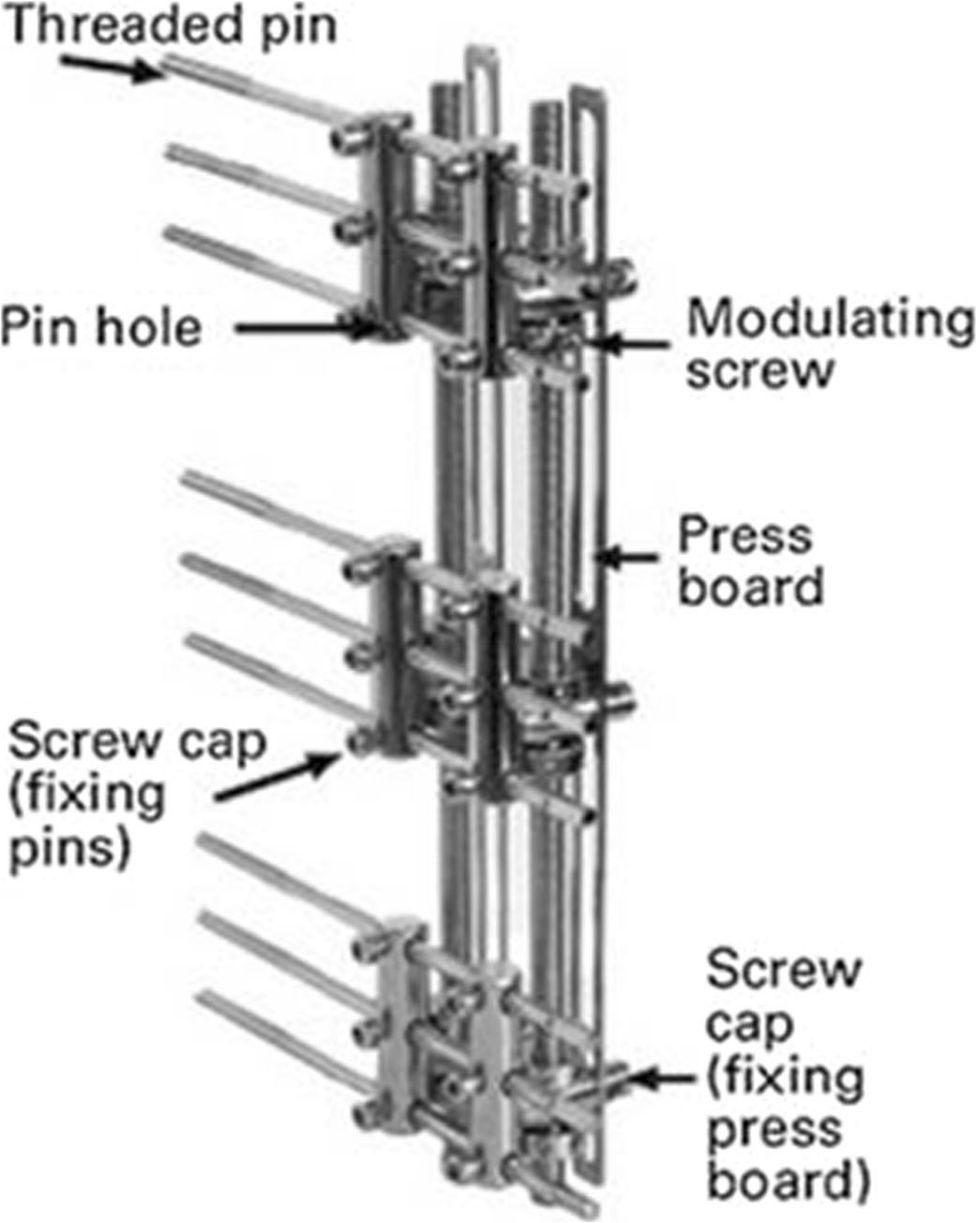
Three-segment monolateral external fixator (Third Medical Instrument Company, Wujin, China)

**Fig. 6:**
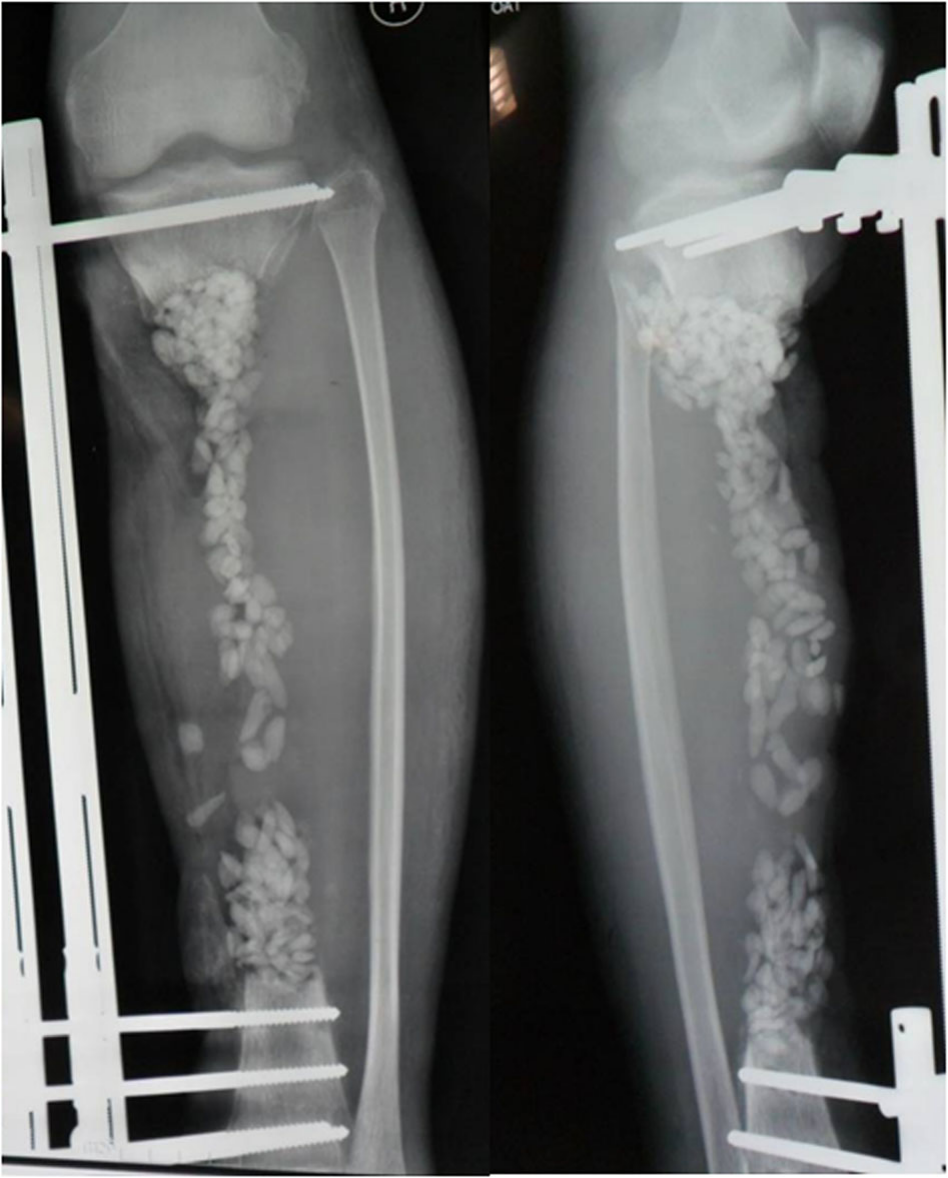
X-ray showing the vancomycin-impregnated cement beads that were placed during the first debridement

Debridement to completely remove the infected and devitalized bone, inflammatory and scar tissue was performed on the second day after admission (Fig. 4). The debrided bone was replaced with a three-segment monolateral external fixator (Third Medical Instrument Company; Fig. 5) followed by the implantation of vancomycin-impregnated cement beads (4 g vancomycin and 80 g cement) in the bone defects (Fig. 6). The wound was partially closed and the dressing was changed regularly following surgery. The second debridement was performed 5 days after the initial procedure and involved the removal of inflammatory tissue and the antibiotic beads. VSD was used to cover the wound and to promote the growth of granulation tissue while simultaneously draining pus. The third debridement was performed 5 days after the second procedure, and involved the removal of the VSD. The wound appeared clean and was subsequently sutured. At this point, the bone defect measured 25 cm in length. Antibiotic sensitivity testing was performed following each of the three debridement procedures and Methicillin-resistant *Staphylococcus aureus*, *Escherichia coli*, *Klebsiella pneumoniae* and *Enterococcus faecalis* were identified. Vancomycin and meropenem through the veins were used to treat the infection. Wound healing and infection control marked the end of the first stage of the treatment plan.

**Fig. 7:**
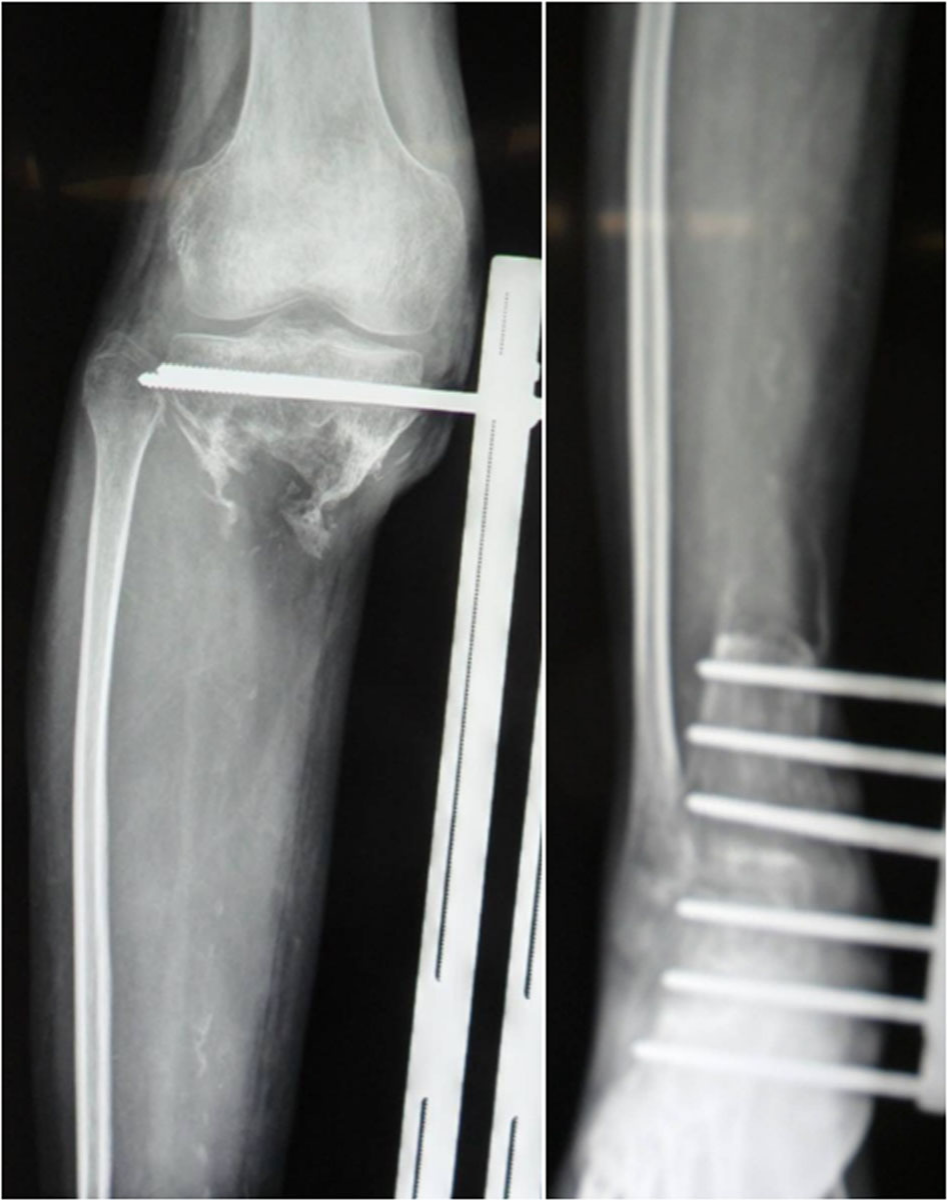
A single osteotomy on the tibia 2 cm away from ankle joint surface was performed

**Fig. 8:**
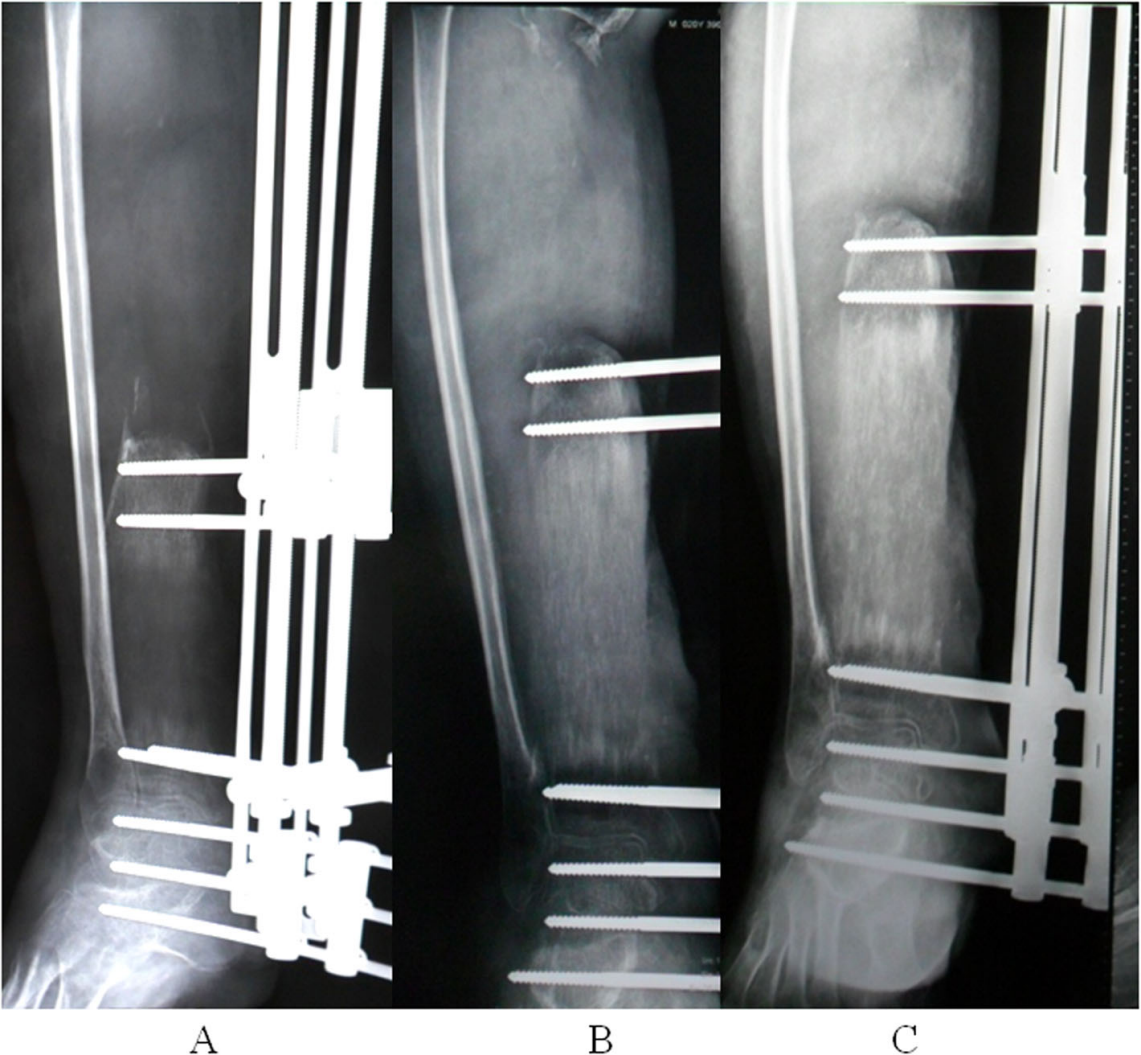
X-ray performed (**a**) 2, (**b**) 4 and (**c**) 6 months after corticotomy demonstrating bone regeneration

**Fig. 9:**
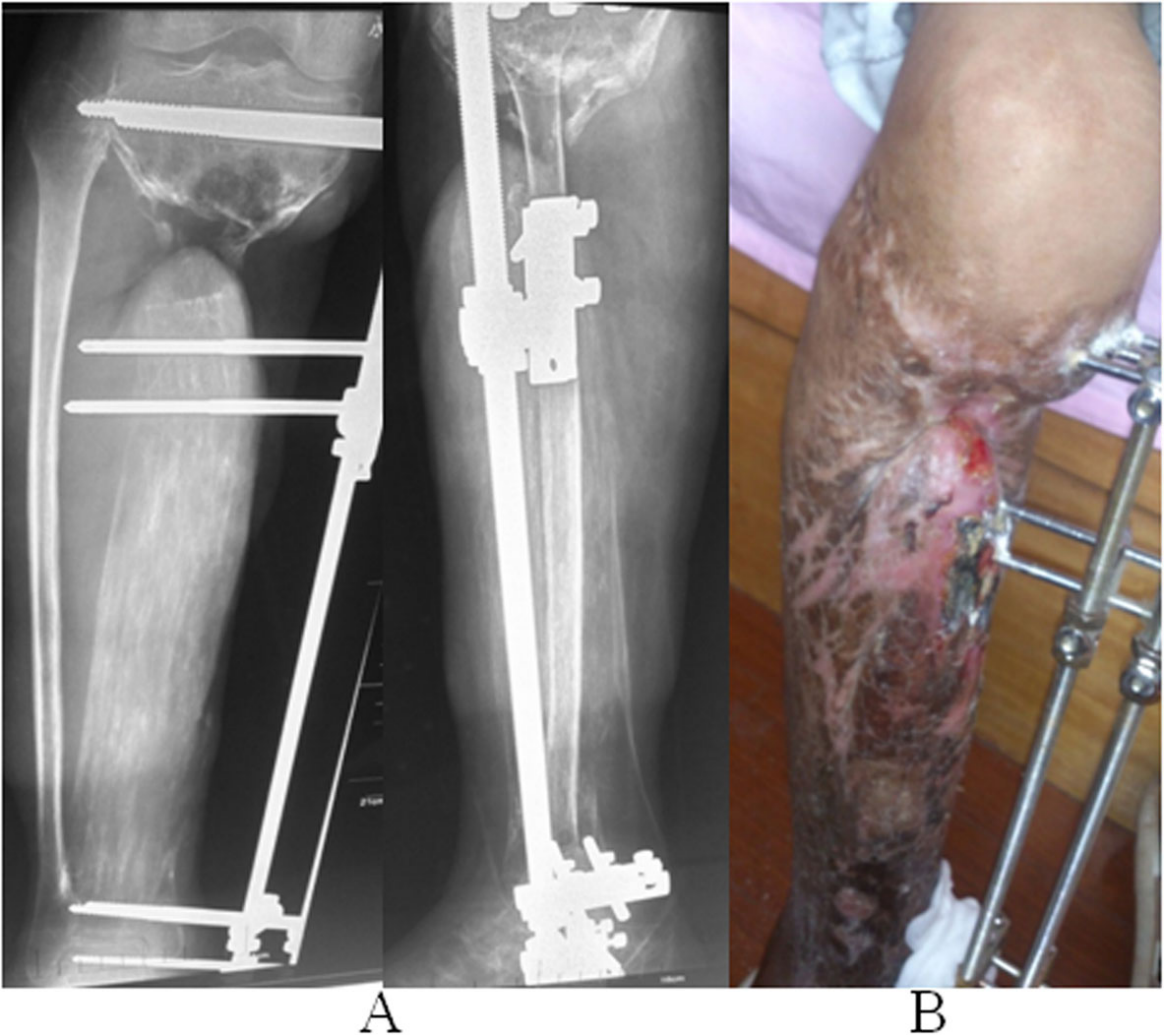
**a** Axis deviation and **b** soft tissue embedding 15 months after corticotomy

**Fig. 10:**
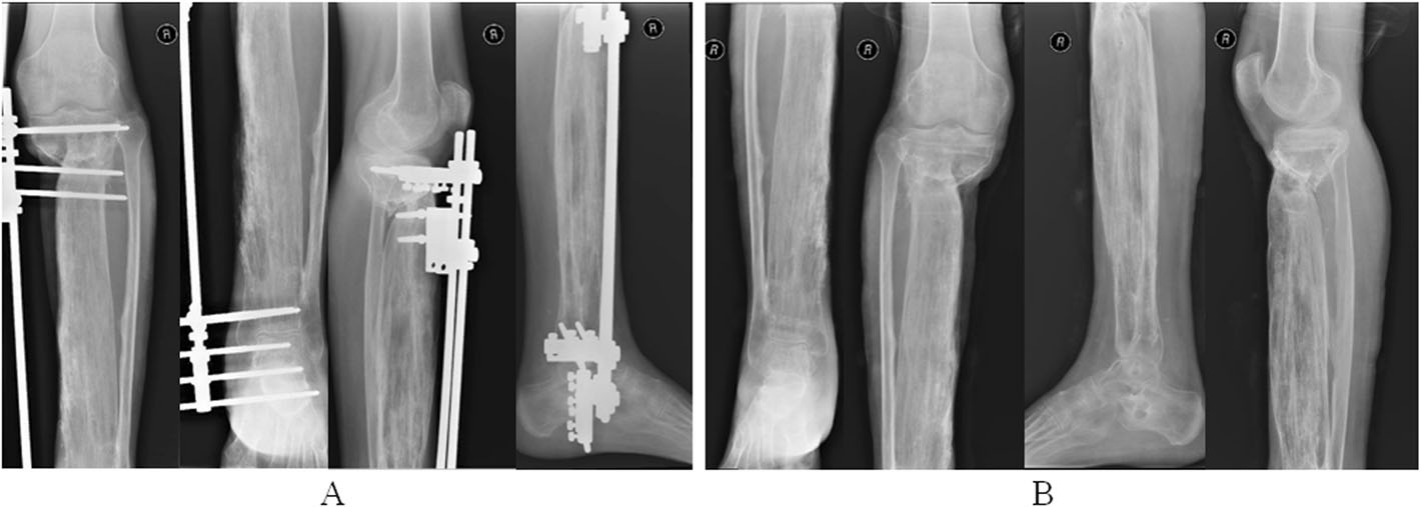
X-ray performed (**a**) 40 months after corticotomy and (**b**) following the removal of the external fixator after complete union at the docking site and bone consolidation

**Fig. 11:**
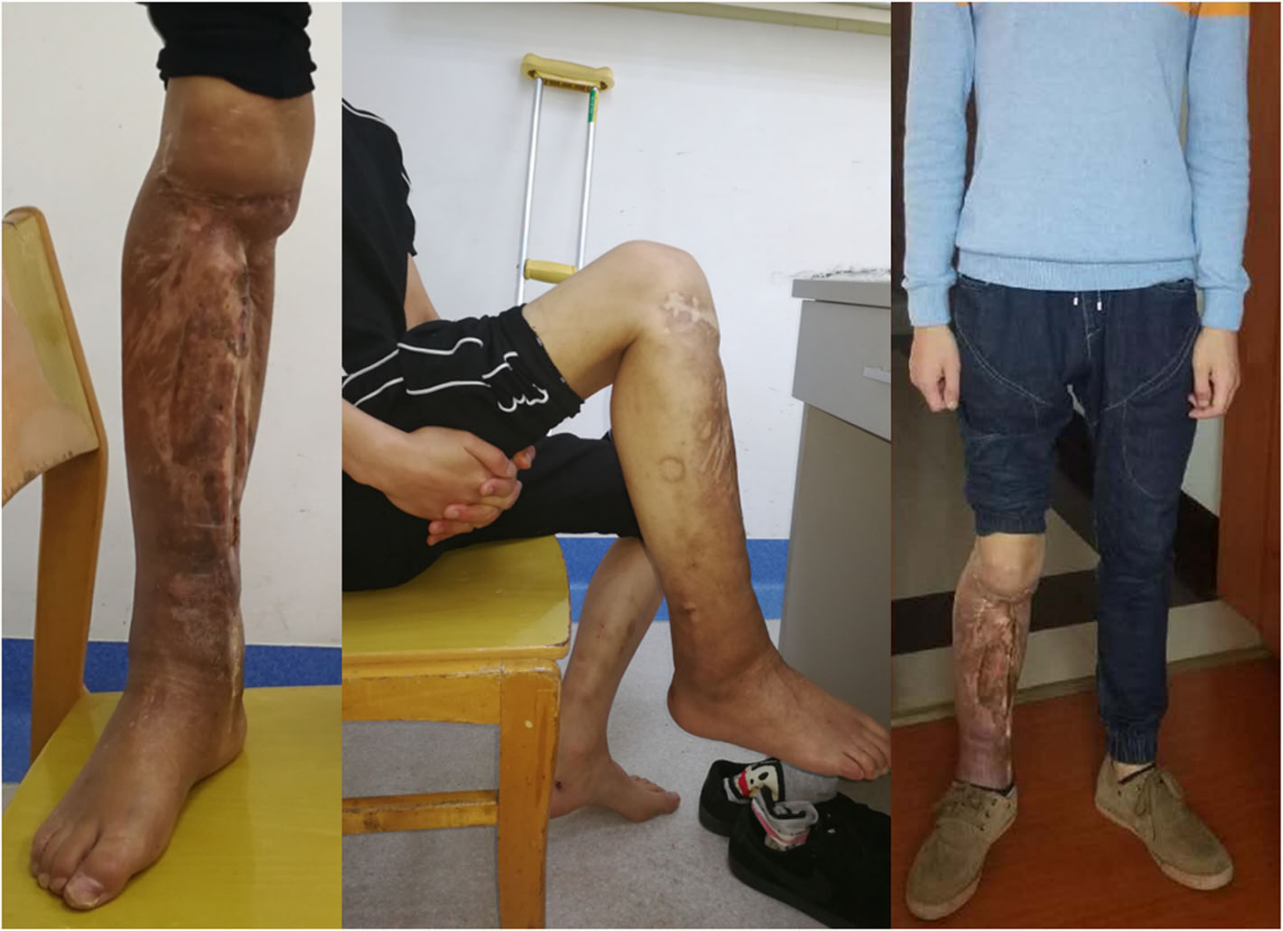
Follow-up after 4 years. Range of motion at the knee was 0–110° and 10° of extension to 10° of flexion at the ankle

The repair of the massive bone defect posed the biggest challenge during the second stage of the treatment plan. As bone transfer technology has a number of advantages, this technique was applied. Wound healing and infection control were assessed through relevant indicators and when satisfactory, a single osteotomy was performed 2 cm away from the ankle joint surface (Fig. 7). Bone transport was initiated 1 week later at a rate of 1 mm per day (0.25 mm four times a day). The quality of the newly formed bone was assessed 2, 4 and 6 months after osteotomy using X-rays (Fig. 8), which revealed satisfactory osteogenesis was occurring. A total of 15 months after osteotomy, the distracted bone had reached docking site, which resulted in skin invagination and axis deviation (Fig. 9). The embedded soft tissue was removed and the axis was adjusted. To promote docking site union, the external fixation was loosened and increasing load across the site of osteogenesis were gradually added. The total length of transport was 24.5 cm. After 40 months, union was achieved and the consolidation of the newly formed bone was complete (Fig. 10). The patient exhibited the following range of motion: i) Knee extension, 0°; ii) knee flexion, 110°; iii) ankle extension, 10°; and iv) knee flexion, 10° (Fig. 11). A knee valgus deformity angle of 4° and a 10 mm shortening of the tibia were observed. The bone healing index was 1.6 months/cm. When the bone and soft tissue wound have healed, the patient was able to walk painlessly without ambulatory assistive devices and resumed daily activities successfully. The ASAMI [12] was used to assess the limb function and bone healing, and revealed a good functional and bone repair result. Similarly, the KSS of functional outcome yielded good result [13] and the LEFS [14] was 65.

Eighteen months after the bone and soft tissue wound have healed, we performed a follow-up evaluation with Short Form-36 (SF-36) score [15] and LEFS (Additional files 1, 2 and 3). The SF-36 score was 86, and the LEFS was 70. The overall treatment progress is briefly presented in Table 1.

**Table 1:** Time line of events from the first debridement performed in our department until removal of the external fixator

Day after admission	Event
Day 2	First debridement (removing the massive bone defect and changing the external fixator).
Day 7	Second debridement.
Day 12	Third debridement (wound closure).
Day 45	Distal tibial corticotomy.
Day 52	Distal tibial distraction was initiated at a rate of 1 mm/day (0.25 mm 4 times/day).
Day 322	The tibia had returned to its original length.
Day 1200	Union at the docking site and consolidation of bone regeneration was achieved. The external fixator was removed.

## Discussion and conclusions

The clinical treatment of massive limb bone defects remains challenging [2, 7, 16] as such injuries require a long treatment period, repeated surgeries and may result in suboptimal outcomes. Furthermore, treatment is associated with high cost and is often accompanied by a high amputation rate [16]. There are currently three main methods to tackle massive bone defects: i) Bone transport using the Ilizarov technique; ii) the Masquelet technique; and iii) bone graft with or without vascular pedicle [16]. The Masquelet technique is controversial, as it requires an autologous bone graft that is difficult to obtain and is associated with recurrent fractures [9]. Furthermore, microsurgical techniques are required. Therefore, the bone transport technique was used to treat bone defects in the present case.

In the present case, the infected bone did not connect due to an open fracture. Following three debridement procedures, the bone defect was 24.5 cm. A large middle bone segment was removed as preoperative imaging examination indicated that this segment was infected and necrotic. Furthermore, no bleeding was occurred in the bone cortex and a large amount of pus was observed in the medullary cavity. The docking site union and consolidation of newly formed bone lasted 25 months. Previous studies revealed that early freshening of the docking site and autologous bone grafting may significantly promote bone union [3, 7, 17–19]. However, in the present case, union was achieved by loosening the external fixation and by the gradual addition of increasing load across the site of osteogenesis.

A bone graft was not performed in the present case for the following reasons: i) The condition of the soft tissue at the docking site was in poor condition and not suitable for bone graft; ii) the docking site was located in the epiphysis region of the tibia, which is served by a rich blood that promotes healing; and iii) the ends of the docking site were wide and union was likely to be obtained through compression according to the authors’ previous experience. However, the time taken for union to occur may have been shortened if an autologous bone graft was performed. Previous studies revealed that that the Ilizarov technique combined with an intramedullary nail or plate may shorten the treatment period and increase patient comfort [1, 3, 20–23]. However, due to high treatment costs and the potential for infection, the internal fixation device was not changed in the patient.

Previous studies revealed that time taken for union to occur may be significantly shortened by double-site osteotomy, which is safe and effective [7, 24]. However, in the present case, the size of the defect shortened the proximal tibia and a double-site osteotomy was not possible. To the best of the authors’ knowledge, the present case report documented the longest bone defect repair performed by single-site corticotomy with bone transport.

16.389ptProblems of axis deviation and soft tissue embedding were encountered during the course of treatment. The axis deviation was corrected by adjusting the external fixation. Surgery was performed to remove the excess soft tissue at the docking site. The complete debridement as well as the removal of the infected and necrotic bone segments in the early stage of treatment were key factors that led to the successful outcome in the present case.

Unilateral and annular external fixations may be used for bone transport [25]. A unilateral external fixation was employed in the present case for the following reasons. A unilateral external fixator is less surgically challenging to attach and can control axis better. Furthermore, a unilateral external fixator facilitates early debridement and is associated with increased patient comfort. Additionally, it has a lower impact on the mobility of the knee joint [16].

Despite the long treatment period and the suboptimal outcome obtained, the patient preferred a limb salvage approach to an amputation and is satisfied with the final result. Furthermore, the total cost of limb salvage is lower than that of amputation [26]. The success of the procedure described in the present case report was a result of the cooperation of the patient and his strong desire to preserve the injured limb as well as the follow-up performed.

As the patient exhibited significantly reduced ankle function, the tibiotalar joint and talocalcaneal joint were attached with a screw when replacing the external fixator. This served to keep the ankle joint in a neutral position and may aid to preserve ankle function and prevent pain and foot drop in later life.

Currently, bone transport is the safest and most effective method for the treatment of massive bone defects. However, this treatment modality requires further optimization as it associated with a number of disadvantages, including a long treatment period, the requirement for repeated surgeries and patient discomfort [9, 16]. Improvements in medical science and technology may allow the development of novel methods that overcome these limitation and improve patient outcomes.

To the best of the authors’ knowledge, the present study described the longest bone defect repair performed using bone transport with single level corticotomy.

## Supplementary information

**Additional file 1.**

**Additional file 2.**

**Additional file 3.**

## Data Availability

All data generated or analyzed during this study are included in this published article.

## Abbreviations

ASAMI: The Association for the study and application of the method of Ilizarov criteria
KSS: Knee society score
LEFS: The lower extremity functional scale
SF-36: Short Form-36
VSD: Vacuum sealing drainage
