# Supplementary figures and images for: Extreme bone lengthening by bone transport with a unifocal tibial corticotomy: a case report
Source: BMC Musculoskelet Disord. 2019 Nov 20;20:555. doi: 10.1186/s12891-019-2927-z (PMC6868736; doi:10.1186/s12891-019-2927-z)

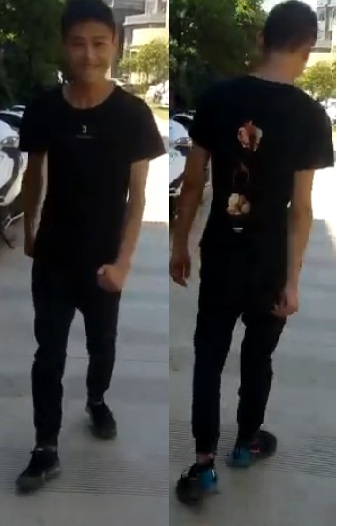

Supplement: Supplementary file 2 — Additional file 2. [file 12891_2019_2927_MOESM2_ESM.png]

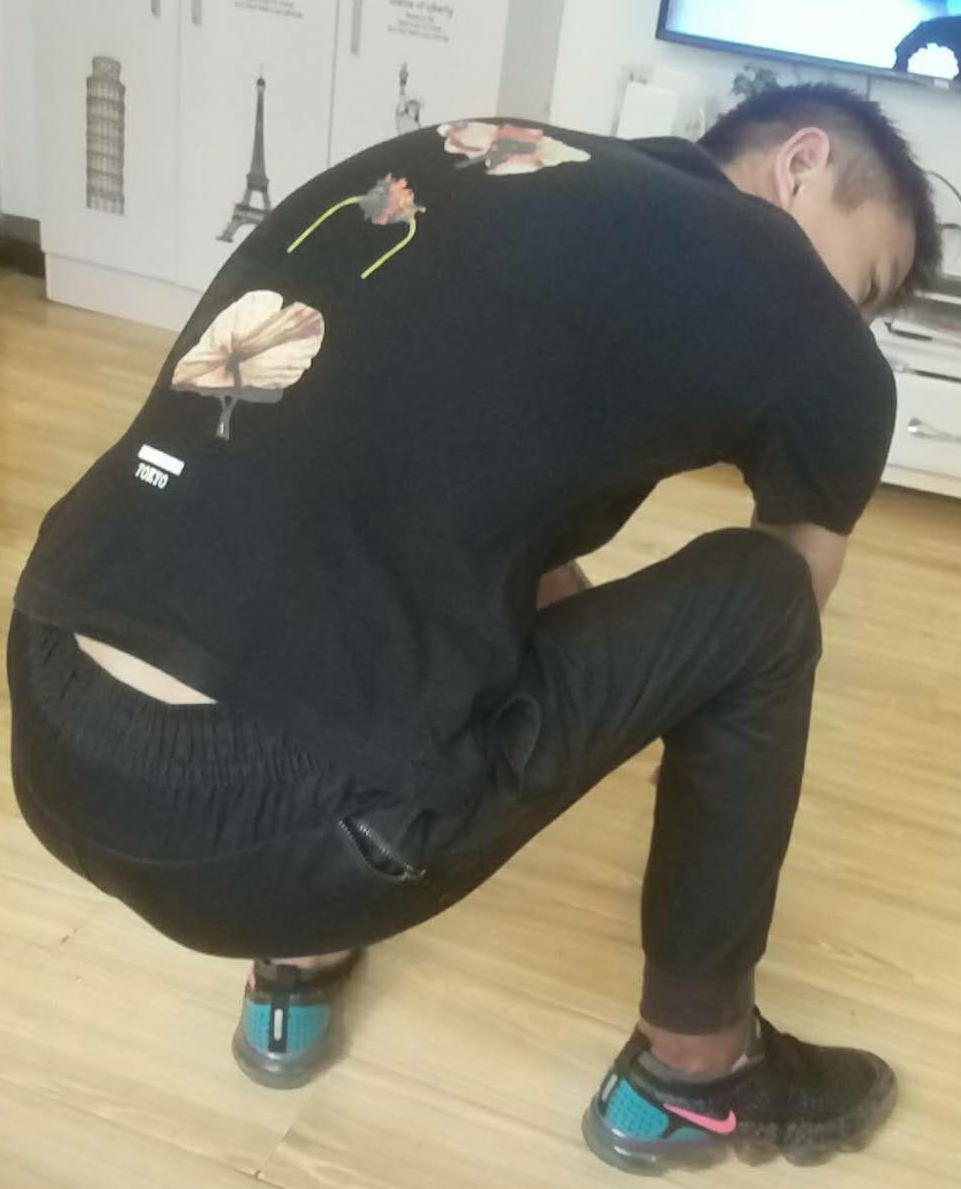

Supplement: Supplementary file 3 — Additional file 3. [file 12891_2019_2927_MOESM3_ESM.tif]
